# How do Dark Triad traits predict workplace deviance? The moderating role of perceived organizational power distance and the mediating role of ingratiation

**DOI:** 10.3389/fpsyg.2026.1937936

**Published:** 2026-09-24

**Authors:** Ayame Tamura

**Affiliations:** Faculty of Law and Letters, Ehime University, Matsuyama, Japan

**Keywords:** Dark Triad traits, ingratiation, mediation analysis, moderation analysis, power distance, workplace deviance

## Abstract

**Background:**

Dark Triad traits have been consistently associated with workplace deviance; however, research examining how organizational conditions shape the expression of these traits is still developing.

**Objectives:**

Drawing on Trait Activation Theory, this study examined whether power distance climate (i.e., an organizational climate characterized by authority, managerial control, and employee obedience) moderates the relationship between the Dark Triad and workplace deviance. Additionally, we examined the mediating effect of ingratiation as a process of the Dark Triad and power distance climate through deviance.

**Methodology:**

A total of 387 (men = 203, Mage = 41.90 ± 10.77) Japanese employees, who completed measures of Dark Triad, power distance climate, ingratiation, workplace bullying, and counterproductive work behavior (CWB) were analyzed.

**Results:**

Hierarchical regression analyses revealed that the main effect of psychopathy and power distance climate on both workplace bullying and CWB was significant. The main effect of Machiavellianism and narcissism on deviance was limited. Furthermore, the interaction between narcissism and power distance climate was significant. A positive association between narcissism and bullying was found under high levels of power distance climate, whereas negative associations between narcissism and both bullying and CWB were observed under low levels of power distance climate. Additionally, the indirect effects of ingratiation through Machiavellianism, narcissism, and power distance climate on workplace bullying were significant. The direct effect of psychopathy on workplace bullying remained in the mediation model.

**Conclusion:**

These findings suggest that perceived organizational environments may facilitate or constrain the expression of the Dark Triad traits, whereas ingratiation may represent an interpersonal factor through which these traits and environments contribute to workplace deviance.

## 1 Introduction

Counterproductive and deviant behaviors in the workplace, such as bullying, aggression, and unethical conduct, refer to voluntary acts that violate organizational norms and harm or are intended to harm organizations and their members (Robinson and Bennett, 1995; Berry et al., 2007). A growing body of research has identified individual personality differences as key antecedents of maladaptive behaviors. The Dark Triad (Paulhus and Williams, 2002)—comprising Machiavellianism, narcissism, and psychopathy—has been consistently associated with negative workplace outcomes. The Dark Triad, characterized by manipulativeness and callous interpersonal tendencies (Jones and Figueredo, 2013), has been linked to a wide range of socially undesirable behaviors, including criminal, delinquent, and aggressive behavior (e.g., Hampejs et al., 2025; Paulhus et al., 2018). These findings indicate that such maladaptive tendencies manifest in organizational settings as reduced job performance and counterproductive work behaviors (e.g., O’Boyle et al., 2012; Sui et al., 2026).

However, accumulating evidence suggests that these behaviors should also be understood within considering the broader organizational context in which they occur (e.g., Czopek, 2026). Organizational climate, defined as employees’ shared perceptions of policies, practices, and procedures (Schneider et al., 2013), exerts a direct influence on a range of employee behaviors and outcomes. In particular, climate captures what behaviors are expected, supported, and rewarded within a given work environment, thereby shaping employees’ behavioral tendencies (Schneider et al., 2013). A growing body of research indicates that organizational climate influences maladaptive workplace behaviors (e.g., Chernyak-Hai and Tziner, 2014; Peterson, 2002), as it provides contextual norms that guide acceptable and deviant conduct. Research on organizational climate has emphasized focused climates, suggesting that specific dimensions of organizational climate should be examined in relation to theoretically relevant outcomes (Schneider et al., 2013). In the present study, we focused on power distance as one such aspect of organizational climate. Power distance refers to the extent to which unequal power distribution is accepted within a social system (Hofstede, 2001). High power distance environments tend to be characterized by authority, managerial control, employee obedience, and status-based interpersonal relationships (Khatri, 2009). Research has shown that power distance is associated with reduced job autonomy, diminished upward communication, less innovation, and greater corruption at the societal level (Daniels and Greguras, 2014). Although power distance originates as a cultural construct, Schneider et al. (2013) argued that organizational climate provides observable manifestations of underlying cultural values and should be understood as being closely intertwined with organizational culture. Furthermore, power distance has been conceptualized at multiple levels of analysis, including the individual, group, organizational, and societal levels (Daniels and Greguras, 2014). Accordingly, the present study focuses on employees’ perceived power distance, operationalized using an organizational climate reflecting power distance characteristic. Throughout the remainder of this manuscript, this climate is referred to as the power distance climate. We examine how the Dark Triad traits and perceptions of this organizational context jointly contribute to workplace deviance.

### 1.1 Interaction between dark triad and power distance climate on workplace deviance

Although Dark Triad traits share certain common features (e.g., Jones and Figueredo, 2013; Paulhus and Williams, 2002), they are conceptually and empirically distinct constructs that differ in the forms and mechanisms of aggressive behavior (e.g., Paulhus et al., 2018). Machiavellianism is characterized by manipulative and exploitative interpersonal strategies (Christie and Geis, 1970) and is associated with strategic forms of indirect aggression for the sake of rewards or social status (e.g., Kerig and Stellwagen, 2010; Malesza, 2018). Psychopathy, in contrast, is defined by emotional callousness, interpersonal egocentricity, and impulsive and antisocial lifestyles (Hare et al., 1990), which show the strongest and most consistent association with severe forms of antisocial behaviors, including violence and criminality (e.g., Hampejs et al., 2025; Leistico et al., 2008). Narcissism is characterized by grandiosity, a strong need for admiration, and a sense of entitlement (Raskin and Hall, 1979); which is generally associated with higher levels of extraverted behaviors (Holtzman et al., 2010), and is also linked to heightened aggression, particularly under conditions of ego threat (e.g., Paulhus et al., 2018; Jones and Paulhus, 2010).

Despite the growing interest in Dark Triad traits in organizational research, relatively few studies have simultaneously examined all three traits in relation to deviant workplace behaviors. Existing evidence suggests that Machiavellianism, psychopathy, and narcissism are positively associated with workplace deviance; however, findings regarding their relative importance remain inconclusive (LeBreton et al., 2018). The most comprehensive evidence comes from O’Boyle et al.’s (2012) meta-analysis, which reported positive associations between Dark Triad traits and counterproductive workplace behaviors (CWB). Interestingly, psychopathy showed a relatively weak bivariate relationship with counterproductive behaviors compared to Machiavellianism and narcissism, a finding that appears inconsistent with the broader literature linking psychopathy to severe forms of antisocial and aggressive behavior. However, subsequent organizational research has continued to demonstrate the maladaptive implications of psychopathy (e.g., Fernández-del-Río et al., 2022). Specifically, a recent meta-analysis focusing on CWB (Duradoni et al., 2025) and workplace bullying (Sui et al., 2026) found that all three Dark Triad traits were positively associated with CWB and bullying perpetration, with psychopathy showing the strongest overall relationship. These findings suggest that although the empirical literature is not entirely consistent, psychopathy remains closely linked to interpersonal and overt forms of antisocial behaviors. Taken together, prior research indicates that all Dark Triad traits contribute to workplace deviance. However, further research is needed to clarify the relative importance of each trait, particularly psychopathy, in predicting workplace deviance.

Importantly, maladaptive behaviors may arise independently of personality traits. Consistent with Trait Activation Theory (Tett and Burnett, 2003), previous research suggests that organizational climates may shape the behavioral expression of the Dark Triad traits. In particular, tendencies of Machiavellianism appear more likely to manifest in deviant and abusive behaviors under climates characterized by low organizational justice (Fernández-del-Río et al., 2022), weak rule enforcement, or a stronger instrumental focus on self-interest and personal gain (De Hoogh et al., 2021). These findings suggest that organizational climates characterized by different norms and expectations may serve as important situational cues for the expression of Dark Triad traits. O’Boyle et al. (2012) found that the relationship between psychopathy and counterproductive work behavior varied depending on job authority, suggesting that factors related to power and organizational hierarchy may also shape the behavioral expression of dark personality traits. In addition, authoritarian leadership has been found to increase workplace bullying (Yang et al., 2025), suggesting that organizational environments emphasizing authority and hierarchy may create conditions that facilitate workplace bullying and other forms of workplace deviance. Given that authority and hierarchy have been identified as important contextual factors associated with workplace deviance, power distance climate may provide a useful framework for understanding how organizational environments shape the expression of Dark Triad traits. It is suggested that environments characterized by high power distance foster tolerance of unethical behaviors because authority figures are less likely to be challenged and unethical conduct is less likely to be exposed (e.g., Daniels and Greguras, 2014; Khatri, 2009). Therefore, this study focuses on power distance climate, characterized by authority, managerial control, and employee obedience. Although previous research has linked hierarchical power structures to the Dark Triad and workplace deviance, these influences have been examined primarily at the individual or interpersonal level. The present study extends this perspective by examining such dynamics as the characteristics of organizational climate.

Drawing on prior findings regarding ethical and justice climates (De Hoogh et al., 2021; Fernández-del-Río et al., 2022), it is considered that maladaptive tendencies of individuals with high Machiavellianism are more likely to manifest in response to organizational cues. Therefore, a climate that reflects power distance may also provide cues that facilitate the expression of Machiavellian tendencies, increasing the likelihood that these individuals will engage in deviant workplace behaviors. Because organizational power, such as job authority, has been associated with workplace deviance (e.g., Lawrence and Robinson, 2007), job authority (occupational category) was included as a control variable in the present study to allow for a more direct examination of the relationships between the Dark Triad traits, power distance climate, and workplace deviance. Therefore, psychopathy is expected to show a more direct relationship with workplace deviance, regardless of the power distance climate. Research on narcissism has similarly demonstrated that organizational settings can shape the behavioral expression of narcissistic tendencies. For example, previous studies have shown that collectivistic cultural environments (O’Boyle et al., 2012) and motivational climate in organization (Braun et al., 2025) can shape the extent to which narcissistic tendencies are translated into workplace behavior. Given that narcissists are strongly motivated to attain status, admiration, and social dominance (Back et al., 2013), an organization characterized by high power distance may provide opportunities for the expression of these tendencies by emphasizing hierarchy and status differentiation. Consequently, such an organizational climate may strengthen the relationship between narcissism and workplace deviance. Collectively, the following hypotheses concerning the interaction between the Dark Triad traits and power distance climate in predicting workplace deviance were established:

*Hypothesis 1*: Machiavellianism and narcissism are positively associated with workplace deviance under high levels of power distance climate. In contrast, no significant interaction will be observed between psychopathy and power distance climate, although the main effect of psychopathy on workplace deviance will remain significant.

### 1.2 The mediating role of ingratiation

One potential mechanism linking both individual traits and organizational context to deviant workplace behavior is ingratiation. Ingratiation is an upward influence strategy, such as communication of direct enhancement by evaluative statements, expressing opinions, or behaving in a manner that is consistent with the target’s stance, doing favors, and self-presentation of one’s own attributes to obtain personal benefits or satisfy organizational goals (Kumar and Beyerlein, 1991). Strategic impression management of ingratiation can facilitate favorable evaluations and interpersonal relationships within organizations (e.g., Kumar and Beyerlein, 1991; Wayne and Liden, 1995), and meta-analytical research suggests that it has positive effects on work outcomes (Higgins et al., 2003). Although ingratiation has traditionally been examined in relation to its functional and adaptive facets, emerging evidence suggests that it may also have negative consequences. Specifically, ingratiatory behavior has been linked to emotional exhaustion, and subsequent deviant workplace behavior (Yan et al., 2020). This may be because ingratiation represents a strategic means rather than a means of building authentic interpersonal relationships.

Importantly, ingratiation was associated with both the Dark Triad traits and power distance climate. Research has shown that individuals with high Dark Triad traits frequently employ strategic interpersonal influence tactics to achieve their personal goals. In particular, psychopathy has been associated with hard tactics (e.g., threat of appeal or punishment), narcissism has been associated with soft tactics (e.g., appearance and offering compliments), and Machiavellianism has been associated with both tactics (Jonason et al., 2012). Based on these findings, Machiavellianism and narcissism, in particular, were considered to be related to ingratiation. Additionally, ingratiation is generally directed toward influential organizational members, such as supervisors and evaluators, who can provide approval and favorable treatment (e.g., Kumar and Beyerlein, 1991; Wayne and Liden, 1995). Furthermore, status hierarchies and power structures have been linked to ingratiatory behavior (e.g., Jones et al., 1963; Pattanayak et al., 2005). Therefore, power distance climate may encourage employees to engage in ingratiatory behavior. Based on the above, the following hypothesis regarding the mediation model of ingratiation is proposed:

*Hypothesis 2*: Machiavellianism and narcissism are positively associated with ingratiatory behavior.

*Hypothesis 3*: Ingratiatory behavior is positively associated with workplace deviance and mediates the relationship between the Dark Triad traits, power distance climate, and workplace deviance.

Although ingratiation is expected to serve as a mechanism linking personality and the organizational climate to workplace deviance, its effects are unlikely to fully account for the emergence of deviant behavior. Therefore, the present study examined both the direct interaction between Dark Triad traits and power distance climate and the indirect pathway through ingratiatory behavior.

In addition to the primary focus on workplace deviance, the present study included a supplementary exploratory examination of annual income as an indicator of individual success to explore whether Dark Triad traits, power distance climate, ingratiatory behavior, and the interactions between Dark Triad traits and power distance climate were associated with income. Previous research on Dark Triad traits has suggested that such traits may benefit individuals while simultaneously harming others (e.g., Jonason et al., 2010). Ingratiation has been conceptualized as a potentially beneficial interpersonal strategy (e.g., Kumar and Beyerlein, 1991; Higgins et al., 2003). Research on Dark Triad traits has also suggested positive associations between narcissism and several adaptive outcomes (e.g., Volmer et al., 2016). Furthermore, certain psychopathic facets may be associated with higher socioeconomic status, including income (e.g., Persson and Lilienfeld, 2019). Therefore, examining income may provide additional insight into whether Dark Triad traits, power distance climate, and their interaction are associated not only with workplace deviance but also with individual success.

## 2 Materials and methods

### 2.1 Participants

Data were collected anonymously via an online survey administered to registered panel members of a commercial survey company in Japan. A screening survey was conducted prior to the main survey and 2,202 responses were obtained. The screening survey targeted men and women in their 20s–50s, in which participants were asked to report their employment status (e.g., full-time employee, part-time or non-regular employee, self-employed/freelancer, unemployed, or preferred not to answer). Based on this screening, individuals who indicated that they were currently working (i.e., full-time, part-time/non-regular, or self-employed/freelancer) were considered eligible. A total of 676 participants (women = 332, mean age = 41.11 ± 19.82) were recruited for the main survey.

Following recommendations to assess careless or insufficient effort responding using multiple detection indicators (e.g., Meade and Craig, 2012; Muszyński, 2023), the responses were screened based on instructed response items, response times, open-ended responses, and consistency with employment-related screening items. Participants were excluded if they failed at least two of the three instructed response items (*N* = 212, 31.36%), provided an inappropriate response to the open-ended age question (*N* = 5, 0.74%), completed the survey in less than half of the median completion time (*N* = 26, 3.85%), or showed more than one inconsistency in their employment-related screening responses (*N* = 111, 16.42%). Cases with a single inconsistency were retained, because such responses were considered likely to reflect incidental response errors. Because these exclusion indicators were not mutually exclusive, some participants met more than one exclusion criterion. After data screening, 387 participants (men = 203, mean age = 41.90 ± 10.77) were retained for analysis. More stringent exclusion criteria (i.e., requiring perfect performance on all attention checks and no screening inconsistencies) produced a broadly similar pattern for the main moderation and mediation analyses (see Supplementary material).

Participation was voluntary and all respondents provided informed consent prior to completing the survey. The participants received incentives from the survey company. This study was approved by the ethics committee of the authors’ institution.

### 2.2 Measures

#### 2.2.1 Dark Triad

The Japanese version of the Short Dark Triad (Jones and Paulhus, 2014; Japanese version: Shimotsukasa and Oshio, 2017) was used to measure Dark Triad traits (Machiavellianism, psychopathy, and narcissism). The Short Dark Triad consisted of 27 items, with each of the three subscales comprising nine items. Example items include “It’s not wise to tell your secrets. (Machiavellianism),” “I am a thrill seeker. (psychopathy),” and “People see me as a natural leader. (narcissism).” Participants rated the items on a five-point scale ranging from 1 (strongly disagree) to 5 (strongly agree).

#### 2.2.2 Power distance climate

The organizational climate reflecting power distance within the organization was assessed using the 12-item Organizational Climate Scale (Fukui et al., 2004), which is a shortened version of the original scale developed by Toshima (1992), drawing on the Work Environment Scale proposed by Moos (1981). This scale has two subscales, Tradition and Organizational Environment, which assess employees’ perception of the organizational environment reflecting managerial assumptions similar to McGregor’s Theory X/Y (Fukui et al., 2004; McGregor, 1960). Hofstede (1980) argued that McGregor’s Theory X/Y can be understood in relation to differences in power distance across cultures. Theory X assumptions have been conceptually linked to high power distance environments characterized by authority, control, and employee obedience (Sorensen and Minahan, 2011; Khatri, 2009). In addition, the content of the dimension emphasizes obedience, managerial control, and traditional organizational norms (Fukui et al., 2004). Accordingly, this dimension was interpreted as reflecting power distance within organizations and was adopted as an indicator of power distance climate in the present study (example item: “It is strictly required that employees follow company policies and regulations”). Although the original scale used a dichotomous response format, to maintain consistency with other work-related scales and to increase response variability (Preston and Colman, 2000), responses were obtained on a 5-point Likert scale ranging from 1: strongly disagree to 5: strongly agree. To examine the measurement properties of this scale using the modified response format, a confirmatory factor analysis was conducted using the weighted least squares mean and variance adjusted (WLSMV) estimator, *χ^2^*(15) = 1520.95, *p* < 0.001, *CFI* = 0.91, *TLI* = 0.85, *RMSEA* = 0.14, 90%*CI* [0.11, 0.16], *SRMR* = 0.06. Robust fit indices are reported throughout. Although the *TLI* and *RMSEA* were not optimal, *CFI* and *SRMR* were within acceptable ranges, and all factor loadings exceeded 0.40 (see Supplementary materials). Because the original scale was designed to assess a broad construct using only a small number of items, its internal consistency was relatively modest (*α* = 0.63). In the present study, the use of the expanded response format was associated with improved internal consistency relative to the original version (*α* = 0.77; see Table 1). Accordingly, these findings support the use of the modified scale in the present study.

**Table 1:** Descriptive statistics and correlations among variables.

		1	2	3	4	5	6	7	8	9	10	11	12	13
Mean		―	41.90	3.77	3.75	―	―	26.17	21.19	19.97	17.70	57.30	29.18	40.34
SD		―	10.77	1.34	2.18	―	―	6.13	6.15	5.94	5.05	19.08	14.99	16.88
Alpha (α)		―	―	―	―	―	―	0.80	0.77	0.80	0.77	0.97	0.98	0.94
1	Gender	―												
2	Age	−0.06	―											
3	Tenure	−0.10	0.37***	―										
4	Income	−0.36***	0.16**	0.29***	―									
5	Manager	−0.23***	0.21***	0.21***	0.51***	―								
6	No system	0.11*	0.06	−0.11*	−0.28***	−0.25***	―							
7	Mac	−0.07	−0.15**	−0.13**	0.02	−0.02	−0.04	―						
8	Psy	−0.29***	−0.30***	−0.11*	0.09	0.05	−0.03	0.47***	―					
9	Nar	−0.21***	−0.23***	−0.05	0.21***	0.17**	−0.06	0.19***	0.61***	―				
10	PDC	−0.14**	−0.07	−0.05	−0.03	−0.07	−0.04	0.30***	0.28***	0.05	―			
11	Ingratiation	−0.09	−0.26***	−0.09	0.09	0.09	−0.07	0.33***	0.40***	0.53***	0.22***	―		
12	Bullying	−0.21***	−0.19***	−0.11*	0.14*	0.08	−0.10	0.33***	0.58***	0.44***	0.22***	0.36***	―	
13	CWB	−0.27***	−0.06	−0.04	0.13*	0.10	−0.11*	0.40***	0.53***	0.27***	0.27***	0.21***	0.77***	―

Manager and no system are dummy-coded variables (non-manager was the reference category). Mac, Machiavellianism; Psy, Psychopathy; Nar, Narcissism; PDC, Power distance; CWB, Counterproductive work behavior.

*p < 0.05, **p < 0.01, ***p < 0.001.

#### 2.2.3 Workplace bullying

Following prior research, workplace deviance was operationalized as CWB, comprising interpersonal and organizational dimensions (e.g., Robinson and Bennett, 1995; Berry et al., 2007; Gruys and Sackett, 2003). Because Dark Triad traits have been consistently associated with interpersonal problems and maladaptive interpersonal behaviors, including workplace bullying (e.g., Paulhus and Williams, 2002; Paulhus et al., 2018; Sui et al., 2026), workplace bullying was assessed alongside the broader CWB measure as a specific indicator of the interpersonal aspect of workplace deviance. It was assessed using the Japanese version of the Negative Acts Questionnaire (Einarsen et al., 2009; Japanese version: Tsuno et al., 2010). As the original scale assessed victimization experiences, the items were adapted to measure perpetration by rephrasing them from the perspective of the perpetrator while retaining the original behavioral content, consistent with approaches used in previous bullying research (e.g., Shaw et al., 2013). For example, “Persistent criticism of your work and effort” was adapted to “Persistently criticizing another person’s work or effort.” Given that this scale includes both a single-item subjective criterion of bullying and 22 objective items assessing the frequency of various negative acts during the previous 6 months, only 22 objective items were used in the present study. Although previous research has proposed multidimensional structures for the Negative Act Questionnaire (e.g., Person- and work-related bullying, Physical or psychological intimidation bullying, and Occupational devaluation; Tsuno et al., 2010), some of these factors are represented by a relatively small number of items. Therefore, this study used the total score as a comprehensive indicator of workplace bullying. Example items include “Having shouted at others or directed sudden anger (or rage).” The items were rated as 1: never, 2 = now and then, 3 = monthly, 4 = weekly, and 5 = daily. To examine the measurement properties of this scale in its adapted form, a confirmatory factor analysis was conducted using the weighted least squares mean and variance adjusted (WLSMV) estimator, *χ^2^*(209) = 290.67, *p* < 0.001, *CFI* = 0.95, *TLI* = 0.95, *RMSEA* = 0.10, 90%*CI* [0.09, 0.12], *SRMR* = 0.02. Although the *RMSEA* was not optimal, which may partly reflect the multidimensional structure proposed in previous research, the remaining fit indices were within acceptable ranges, all factor loadings exceeded 0.40 (see Supplementary materials), and internal consistency was acceptable (*α* = 0.78; see Table 1). Consistent with the rationale described above, the total score was used as a comprehensive indicator of workplace bullying.

#### 2.2.4 Counterproductive workplace behavior

The Japanese Organizational Dysfunctional Behavior Scale (Tanaka and Toshima, 2005) was used to assess CWB comprehensively. This scale was developed by selecting items based on existing measures of behaviors that impair organizational functioning as well as findings from interview-based investigations of behaviors observed in actual Japanese organizational and workplace settings (Tanaka and Toshima, 2005). Factor analysis identified four dimensions: serious dysfunctional behavior, sabotage, verbal harassment, and verbal violence. Consistent with the workplace deviance framework proposed by Robinson and Bennett (1995), these dimensions capture a broad range of dysfunctional workplace behaviors (Tanaka and Toshima, 2005). To avoid an excessive number of dependent variables and assess overall dysfunctional workplace behavior, a total score was used in the present study. Example items include “Damaging shared property in the company or workplace, resulting in losses.” Participants were asked to reflect on their recent workplace situations and indicate whether they had engaged in each behavior using a 5-point scale with 1 = have never thought about doing it, 2 = have thought about doing it but never carried it out, 3 = once or twice, 4 = a few times, and 5 = frequently.

#### 2.2.5 Ingratiation

To assess ingratiatory behavior, the Measures of Ingratiating Behavior in Organizational Setting (Kumar and Beyerlein, 1991) was translated into Japanese with permission. This scale consists of 24 items and has four subscales: other enhancement, opinion conformity, self-presentation, and favor rendering. Example items include “Impress upon your supervisor that only he/she can help you in a given situation mainly to make him/her feel good about himself/herself (other enhancement).” The items are rated as 1: never do it; 2 = seldom do it; 3 = occasionally do it; 4 = often do it; and 5 = almost always do it. To examine the measurement properties of the Japanese translation of this scale, a confirmatory factor analysis with the WLSMV was conducted (*χ^2^*(246) = 745.88, *p* < 0.001, *CFI* = 0.93, *TLI* = 0.92, *RMSEA* = 0.08, 90%*CI* [0.08, 0.09], *SRMR* = 0.03). Robust fit indices are reported throughout. Although the *RMSEA* was not optimal, the remaining fit indices were within acceptable ranges, and all factor loadings exceeded 0.40 (see Supplementary materials). Therefore, the four-factor structure of the Japanese version was considered acceptable. Internal consistencies were high (*α*s = 0.87–0.92), and moderate-to-high correlations with political skill provided additional evidence of construct validity (*rs* = 0.48–0.70). Taken together, these findings suggest that the Japanese version retained the measurement properties of the original scale. Given the substantial inter-factor correlations (*r*s = 0.92–0.98), a composite total score was used in the main analyses. The detailed results are reported in the Supplementary materials.

#### 2.2.6 Political skill

To validate translated Measures of Ingratiating Behavior in Organizational Setting, Political Skill Inventory (Ferris et al., 2005) of the Japanese version (Ohshima et al., 2016) was administered. This scale consists of 18 items and four subscales: social astuteness, interpersonal influence, networking ability, and apparent sincerity. Example items include “I spend a lot of time and effort at work networking with others (networking ability).” Participants rated the items on a 7-point scale ranging from 1 (strongly disagree) to 7 (strongly agree). Although related to ingratiation, political skill reflects a broader interpersonal influence capability (Ferris et al., 2005), whereas ingratiation refers to specific interpersonal tactics aimed at increasing attractiveness and obtaining favorable outcomes from others (Kumar and Beyerlein, 1991). Because the present study focused on conformity and ingratiatory behaviors within organizational hierarchies, ingratiation was considered more directly relevant to the proposed theoretical model. Therefore, only ingratiation was included in the primary analyses, and political skill was excluded.

#### 2.2.7 Other variables

As control variables, gender (1 = men, 2 = women), age, job tenure (1 = less than 1 year, 2 = 1–3 years, 3 = 3–5 years, 4 = 5–10 years, 5 = 10 years or more), and occupational category (manager, non-manager, and no formal system = absence of a formal organizational or management structure) were measured. The occupational category was dummy-coded with non-managers as the reference group (1 = manager, 0 = otherwise; 1 = no formal system, 0 = otherwise). Among the participants, 55 (19%) were managers, 167 (58%) were non-managers, and 61 (21%) worked in organizations without formal structures. Responses of “prefer not to answer” (*N* = 5) were treated as missing values. Furthermore, as a supplemental outcome index reflecting the adaptive facet of the workplace, annual income was measured as follows 1: under 2 million yen, 2:2–2.99 million yen, 3:3–3.99 million yen, 4:4–4.99 million yen, 5:5–5.99 million yen, 6:6–7.99 million yen, 7:8–9.99 million yen, 8:10 million yen or more; 9, prefer not to answer; 10, other. Responses 9: Prefer not to answer and 10: Others were treated as missing variables. Income was treated as an ordinal variable because these categories represent increasing levels of income.

### 2.3 Data analysis

Analyses were conducted to test the hypothesized moderating effects and the mediation model. Regarding moderating effects, we examined whether the interaction between the Dark Triad traits and power distance climate significantly predicted deviant workplace behaviors. In the hierarchical multiple regression analysis, control variables (i.e., gender, age, tenure, and occupational categories dummy-coded as “manager” and “formal system,” with “non-manager” serving as the reference group) were entered in Step 1. The explanatory variables (i.e., each trait of the Dark Triad and power distance climate) were entered in Step 2, and their interaction terms were entered in Step 3. To determine whether the interaction between the Dark Triads and power distance climate on workplace deviance could be observed through a process distinct from the mediation model, which was subsequently analyzed, ingratiation was included in Steps 2 and 3. These analyses were conducted separately for each outcome variable (workplace bullying and CWB). Additionally, supplementary analyses predicting income were conducted using hierarchical logistic regression because income was treated as an ordinal variable. When a significant interaction was observed, simple slope tests were conducted to examine further interaction terms, with power distance climate as the moderator, and the interaction probed at the mean and one standard deviation above and below the mean. In addition, the Johnson–Neyman technique was employed to determine the range of moderator values at which the simple effect became statistically significant.

In the mediation model, we examined whether the Dark Triad traits and power distance climate were positively associated with ingratiation, which in turn was associated with deviant workplace behaviors. It was first confirmed that Dark Triad traits and power distance climate were significantly associated with ingratiation (X → M). In the hierarchical regression analysis of Step 2, because it was already examined whether ingratiation was significantly associated with deviant workplace behaviors (M → Y) after controlling for both independent variables, the results refer to the prior regression analysis, and no repetitive analysis was conducted. For those outcome variables in which both paths (X → M and M → Y) were significant, indirect effects were estimated using structural equation modeling (SEM) with bootstrapping (5,000 resamples). Although workplace bullying represents the interpersonal aspect of workplace deviance, the CWB measure used in the present study captures broader dysfunctional workplace behaviors and includes multiple interpersonal and organizational elements. Accordingly, in the SEM analysis, indirect effects were estimated separately for each outcome, rather than using a single latent variable to represent workplace deviance.

The statistical procedures were performed with R version 4.4.1 (R Core Team, 2024).

## 3 Results

Descriptive statistics and Pearson’s correlation coefficients for all variables are presented in Table 1.

### 3.1 Moderation analysis

The results of the hierarchical multiple regression analyses predicting deviant workplace behaviors (workplace bullying and CWB), including the interaction terms between the Dark Triad traits and power distance climate, are presented in Tables 2 and 3. Regarding the regression prediction of workplace bullying (Table 2), psychopathy, narcissism, and power distance climate showed significant positive main effects in Step 3. As shown in Table 3, Machiavellianism, psychopathy, and power distance climate had significant positive effects on CWB in Step 3. In addition to the main effect of each variable, the focal predictors in Step 2 explained an additional 26–27% of the variance in the outcome variables.

**Table 2:** Results of hierarchical regression analyses predicting workplace bullying.

	Step 1					Step 2					Step 3				
	*β*	*B*	SE	*p*	sr^2^	*β*	*B*	SE	*p*	sr^2^	*β*	*B*	SE	*p*	sr^2^
Gender	−0.20	−6.01	1.52	0.00	0.04	−0.04	−1.19	1.34	0.38	0.00	−0.02	−0.59	1.29	0.65	0.00
Age	−0.19	−0.27	0.08	0.00	0.03	0.01	0.02	0.07	0.83	0.00	−0.02	−0.02	0.07	0.72	0.00
Tenure	−0.08	−0.93	0.62	0.13	0.01	−0.06	−0.70	0.52	0.18	0.00	−0.04	−0.48	0.50	0.34	0.00
Manager	0.08	2.84	2.01	0.16	0.01	0.02	0.87	1.73	0.62	0.00	0.04	1.62	1.66	0.33	0.00
No system	−0.05	−1.99	1.92	0.30	0.00	−0.07	−2.42	1.62	0.14	0.00	−0.06	−2.18	1.56	0.16	0.00
Ingratiation						0.10	0.07	0.04	0.07	0.01	0.05	0.04	0.04	0.34	0.00
Mac						0.05	0.13	0.12	0.30	0.00	0.02	0.05	0.12	0.69	0.00
Psy						0.42	1.03	0.15	0.00	0.08	0.41	1.00	0.15	0.00	0.07
Nar						0.10	0.25	0.15	0.09	0.01	0.11	0.27	0.14	0.06	0.01
PDC						0.06	0.18	0.14	0.19	0.00	0.10	0.31	0.13	0.02	0.01
Mac*PDC											0.07	0.03	0.02	0.16	0.00
Psy*PDC											0.09	0.04	0.03	0.14	0.00
Nar*PDC											0.16	0.08	0.02	0.00	0.02
*R^2^*	0.09					0.36					0.42				
*ΔR^2^*	0.09					0.27					0.06				

Manager and No system are dummy-coded variables (non-manager was the reference category). Mac, Machiavellianism; Psy, Psychopathy; Nar, Narcissism; PDC, Power distance climate; CWB, Counterproductive work behavior.

**Table 3:** Results of hierarchical regression analyses predicting counterproductive work behavior.

	Step 1					Step 2					Step 3				
	*β*	*B*	SE	*p*	sr^2^	*β*	*B*	SE	*p*	sr^2^	*β*	*B*	SE	*p*	sr^2^
Gender	−0.25	−8.47	1.72	0.00	0.06	−0.10	−3.51	1.54	0.02	0.01	−0.09	−3.10	1.52	0.04	0.01
Age	−0.07	−0.10	0.09	0.23	0.00	0.09	0.14	0.08	0.08	0.01	0.07	0.11	0.08	0.16	0.00
Tenure	−0.06	−0.75	0.70	0.29	0.00	−0.03	−0.39	0.60	0.51	0.00	−0.02	−0.23	0.59	0.70	0.00
Manager	0.05	2.15	2.28	0.35	0.00	0.04	1.88	1.99	0.34	0.00	0.06	2.46	1.95	0.21	0.00
No system	−0.07	−2.89	2.18	0.19	0.00	−0.07	−3.06	1.86	0.10	0.01	−0.07	−3.02	1.84	0.10	0.01
Ingratiation						−0.03	−0.03	0.05	0.57	0.00	−0.06	−0.06	0.05	0.23	0.00
Mac						0.18	0.51	0.14	0.00	0.02	0.16	0.45	0.14	0.00	0.02
Psy						0.44	1.21	0.17	0.00	0.09	0.43	1.18	0.17	0.00	0.08
Nar						−0.04	−0.11	0.17	0.52	0.00	−0.03	−0.10	0.17	0.57	0.00
PDC						0.09	0.31	0.16	0.05	0.01	0.12	0.42	0.16	0.01	0.01
Mac*PDC											0.08	0.03	0.02	0.12	0.00
Psy*PDC											0.04	0.02	0.03	0.51	0.00
Nar*PDC											0.11	0.06	0.03	0.03	0.01
*R^2^*	0.07					0.33					0.36				
*ΔR^2^*	0.07					0.26					0.03				

Manager and No system are dummy-coded variables (non-manager was the reference category). Mac, Machiavellianism; Psy, Psychopathy; Nar, Narcissism; PDC, Power distance climate; CWB, Counterproductive work behavior.

Furthermore, the interaction between narcissism and power distance climate was significant in models with both workplace bullying and CWB as outcome variables (Tables 2 and 3). As depicted in Figure 1, narcissism was positively associated with workplace bullying when the power distance climate was one standard deviation above the mean (*b* = 0.65, *SE* = 0.18, *p* < 0.001), whereas this association was not significant when the power distance climate was the mean (*b* = 0.27, *SE* = 0.14, *p* = 0.06) and one standard deviation below the mean (*b* = −0.12, *SE* = 0.19, *p* = 0.52). The Johnson–Neyman analysis further revealed a small region of significance at very low levels of power distance climates (Figure 2). Additionally, as depicted in Figure 3, narcissism was marginally negatively associated with CWB when the power distance climate was one standard deviation below the mean (*b* = −0.40, *SE* = 0.22, *p* = 0.07), whereas this association was not significant when the power distance climate was at the mean (*b* = −0.10, *SE* = 0.17, *p* = 0.55) and one standard deviation above the mean (*b* = 0.20, *SE* = 0.21, *p* = 0.36). The Johnson–Neyman plot is also presented in Figure 4.

**Figure 1:**
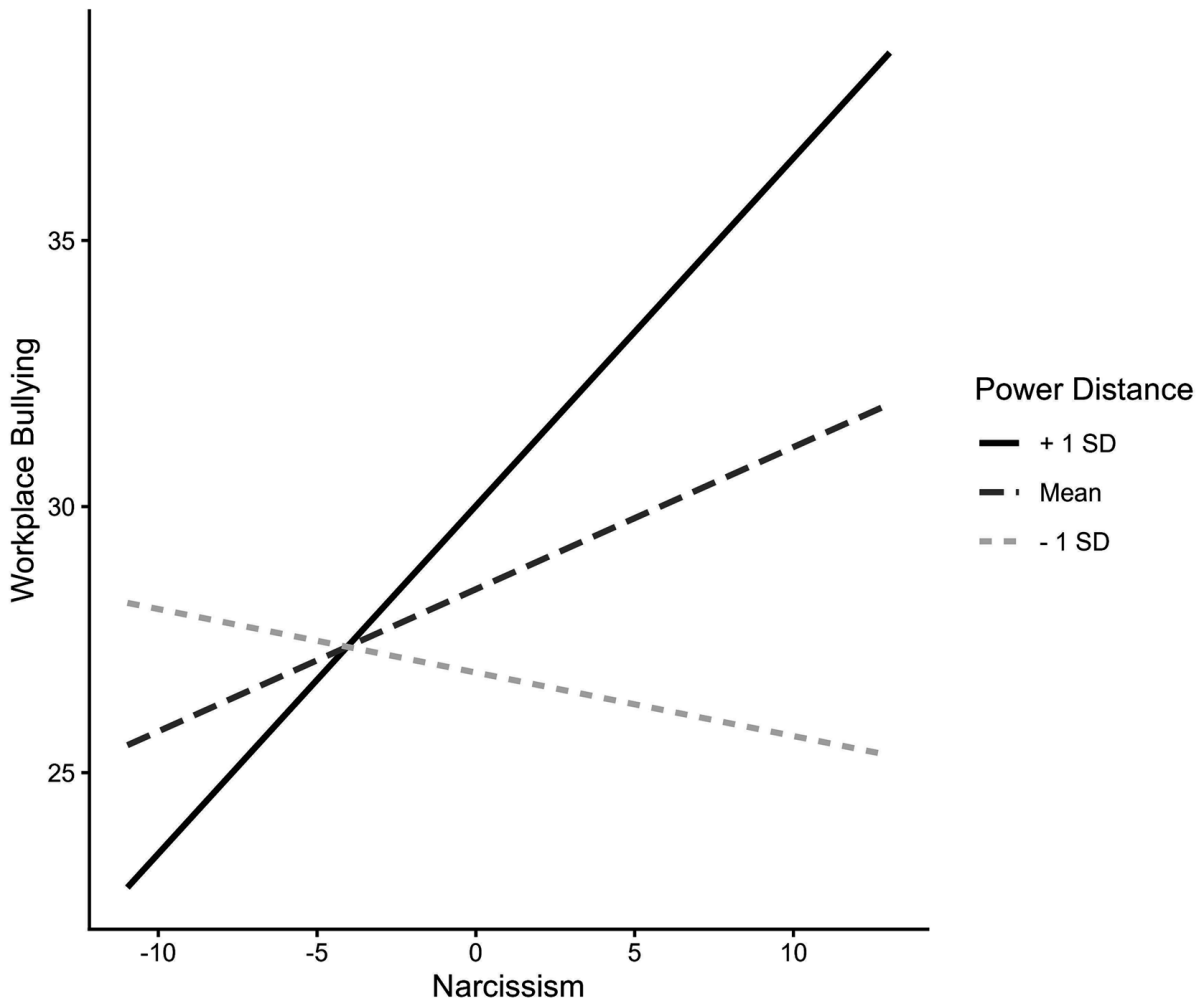
Simple slope analysis of the interaction between narcissism and power distance predicting workplace bullying.

**Figure 2:**
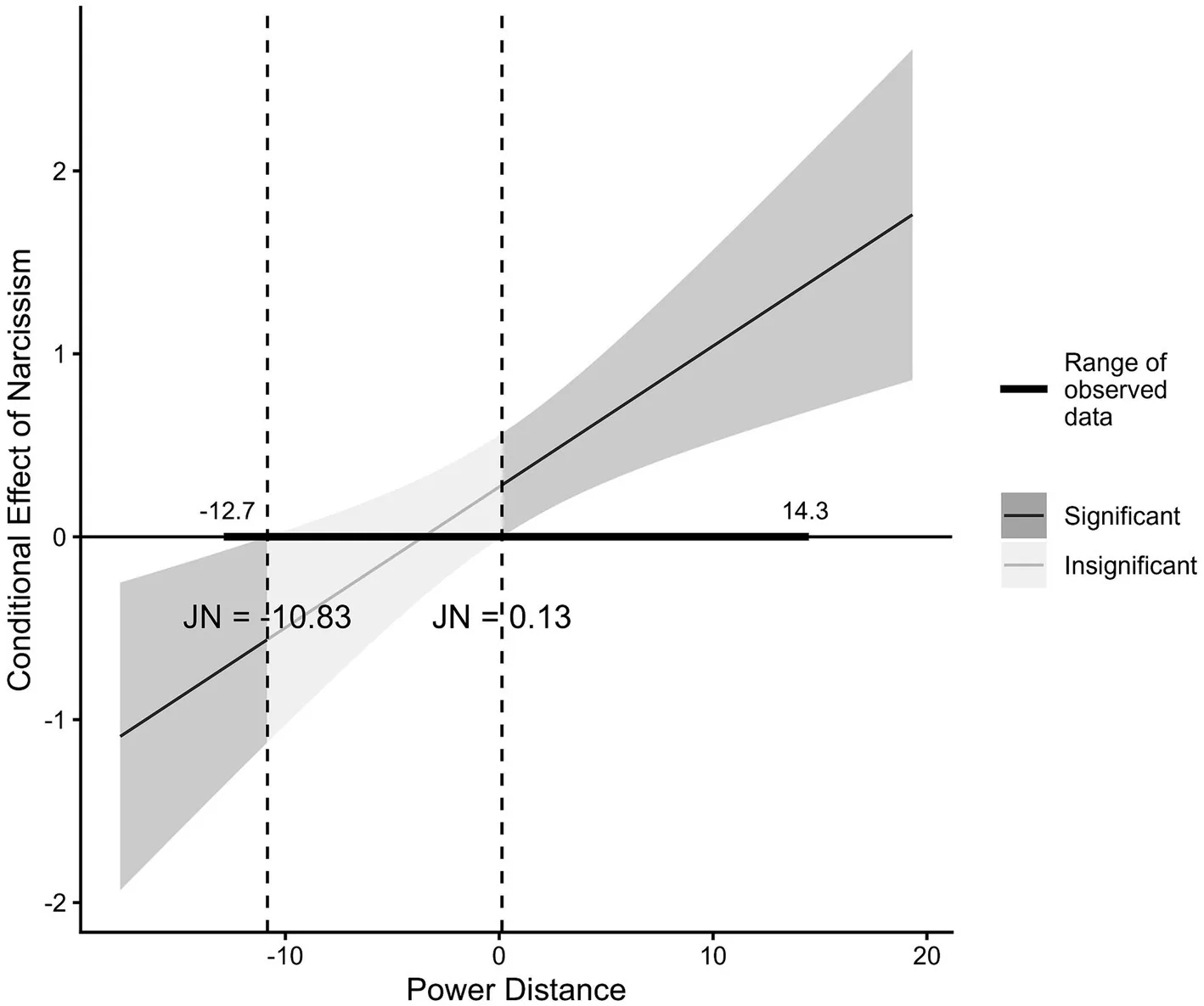
Johnson–Neyman plot showing the regions of significance for the conditional effect of narcissism on workplace bullying.

**Figure 3:**
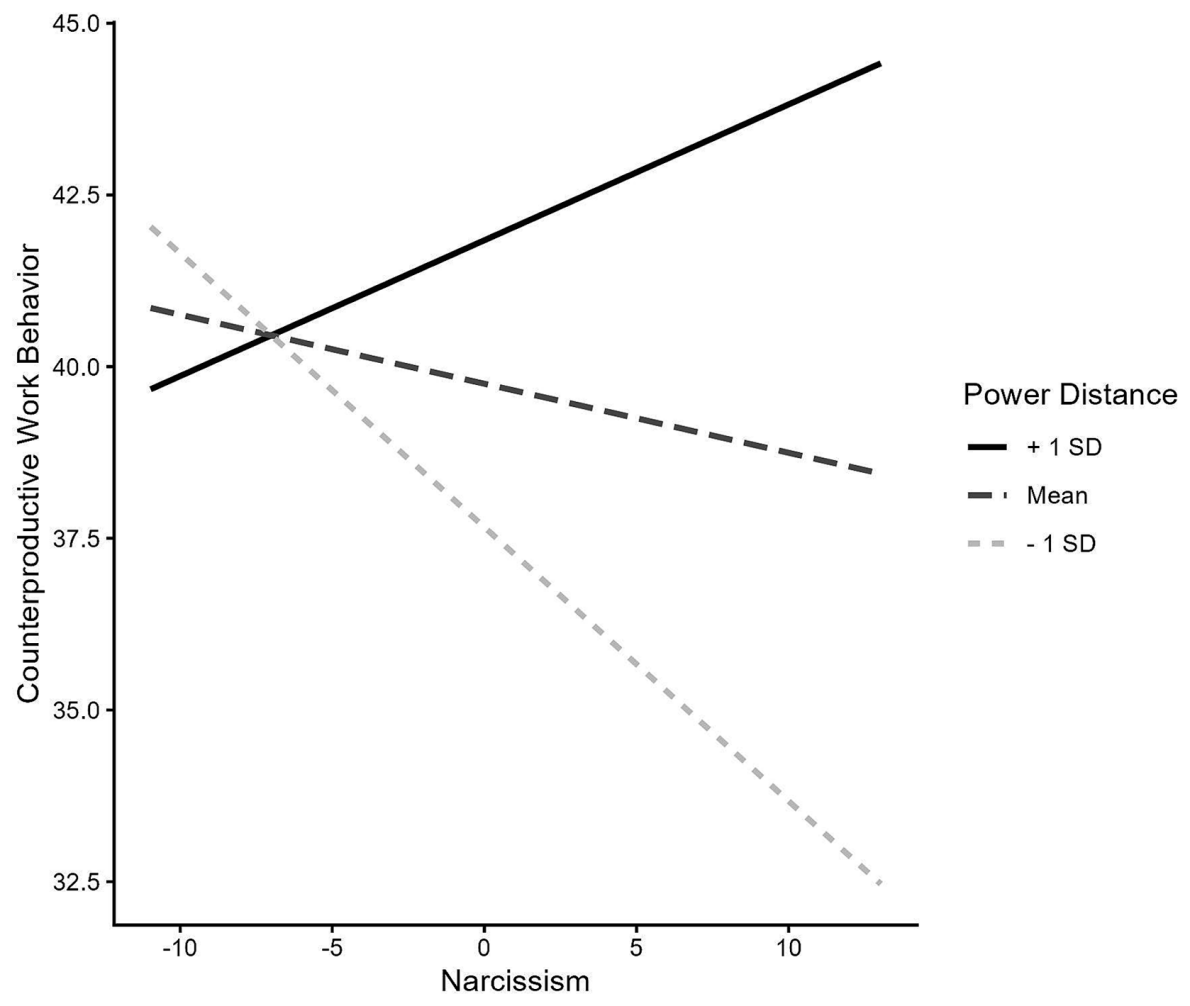
Simple slope analysis of the interaction between narcissism and power distance predicting counterproductive work behavior.

**Figure 4:**
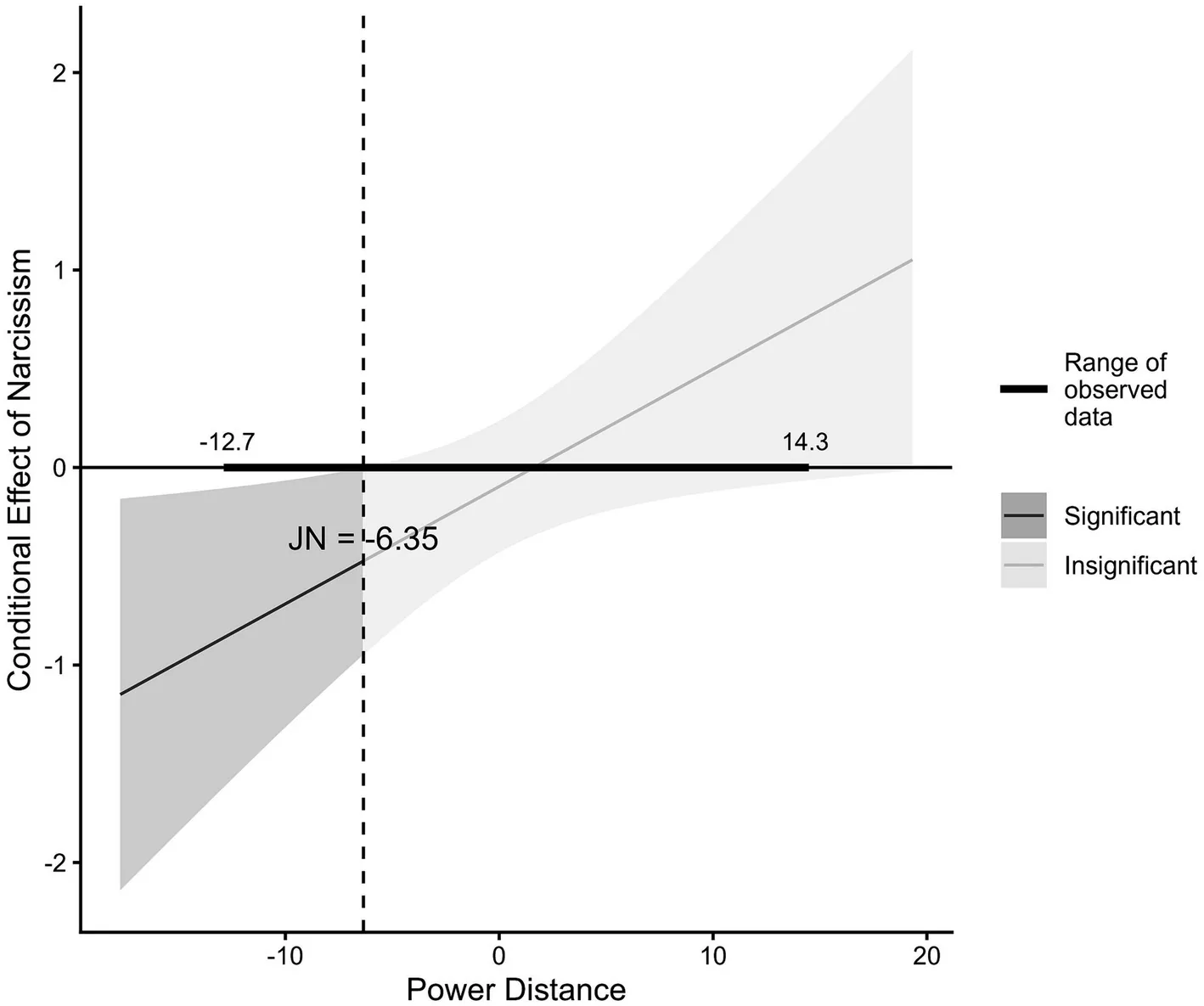
Johnson–Neyman plot showing the regions of significance for the conditional effect of narcissism on counterproductive work behavior.

The results of the supplementary hierarchical logistic regression analyses for income prediction are presented in Table 4. Only the main effect of narcissism was significant but small, and no other main effects or interactions were observed.

**Table 4:** Results of hierarchical ordinal logistic regression predicting annual income.

	Step 1					Step 2					Step 3				
	*B*	SE	OR	95%CI:		*B*	SE	OR	95%CI:		*B*	SE	OR	95%CI:	
				Lower	Upper				Lower	Upper				Lower	Upper
Gender	−1.03	0.21	0.36	0.24	0.53	−0.99	0.22	0.37	0.24	0.57	−1.00	0.22	0.37	0.24	0.57
Age	0.00	0.01	1.00	0.98	1.02	0.00	0.01	1.00	0.98	1.02	0.00	0.01	1.00	0.98	1.02
Tenure	0.24	0.08	1.27	1.08	1.49	0.25	0.08	1.29	1.10	1.52	0.25	0.08	1.28	1.09	1.51
Manager	2.01	0.28	7.43	4.30	13.04	1.92	0.29	6.83	3.90	12.15	1.91	0.29	6.76	3.86	12.04
No system	−1.07	0.28	0.34	0.19	0.60	−1.08	0.29	0.34	0.19	0.59	−1.08	0.29	0.34	0.19	0.60
Ingratiation						0.00	0.01	1.00	0.98	1.01	0.00	0.01	1.00	0.98	1.01
Mac						0.02	0.02	1.02	0.99	1.06	0.03	0.02	1.03	0.99	1.07
Psy						−0.01	0.02	0.99	0.95	1.04	−0.01	0.02	0.99	0.95	1.04
Nar						0.05	0.02	1.05	1.00	1.10	0.05	0.02	1.05	1.00	1.10
PDC						−0.02	0.02	0.98	0.94	1.02	−0.02	0.02	0.98	0.94	1.02
Mac*PDC											0.00	0.00	1.00	0.99	1.01
Psy*PDC											0.00	0.00	1.00	0.99	1.01
Nar*PDC											0.00	0.00	1.00	0.99	1.01
*McFadden’s R^2^*	0.11					0.11					0.11				
*ΔR^2^*	0.11					0.00					0.00				

Manager and No system are dummy-coded variables (non-manager was the reference category). Mac, Machiavellianism; Psy, Psychopathy; Nar, Narcissism; PDC, Power distance climate; CWB, Counterproductive work behavior.

### 3.2 Mediation model

Additional regression analysis was conducted to examine whether Dark Triad traits and power distance climate were associated with ingratiation (Table 5). The results showed that all the Dark Triad traits and power distance climate were significantly associated with ingratiation. However, in prior Step 2 of the hierarchical regression model predicting workplace bullying and CWB (Table 2 and Table 3), ingratiation was marginally associated with workplace bullying (*β* = 0.10, *B* = 0.07, *SE* = 0.04, *p* = 0.07) but was unrelated to CWB (*β* = −0.03, *B* = −0.03, *SE* = 0.05, *p* = 0.57). Therefore, indirect effects were estimated only for workplace bullying.

**Table 5:** Multiple regression analyses predicting ingratiation.

	*β*	*B*	SE	*p*	sr^2^
Gender	0.04	1.42	1.70	0.40	0.00
Age	−0.13	−0.24	0.08	0.01	0.01
Tenure	−0.02	−0.26	0.66	0.69	0.00
Manager	0.07	3.36	2.19	0.13	0.00
No system	−0.01	−0.51	2.05	0.80	0.00
Mac	0.21	0.66	0.15	0.00	0.03
Psy	−0.10	−0.31	0.19	0.11	0.00
Nar	0.50	1.60	0.17	0.00	0.15
PDC	0.18	0.70	0.17	0.00	0.03
*R^2^*	0.36				

Manager and No system are dummy-coded variables (non-manager was the reference category). Mac, Machiavellianism; Psy, Psychopathy; Nar, Narcissism; PDC, Power distance climate.

SEM with bootstrapped confidence intervals was used to test the hypothesized mediation effects (Figure 5 and Table 6). Significant indirect effects of ingratiation were observed for all Dark Triad traits except psychopathy and power distance climate in predicting workplace bullying, although the magnitudes of these indirect effects were generally small.

**Figure 5:**
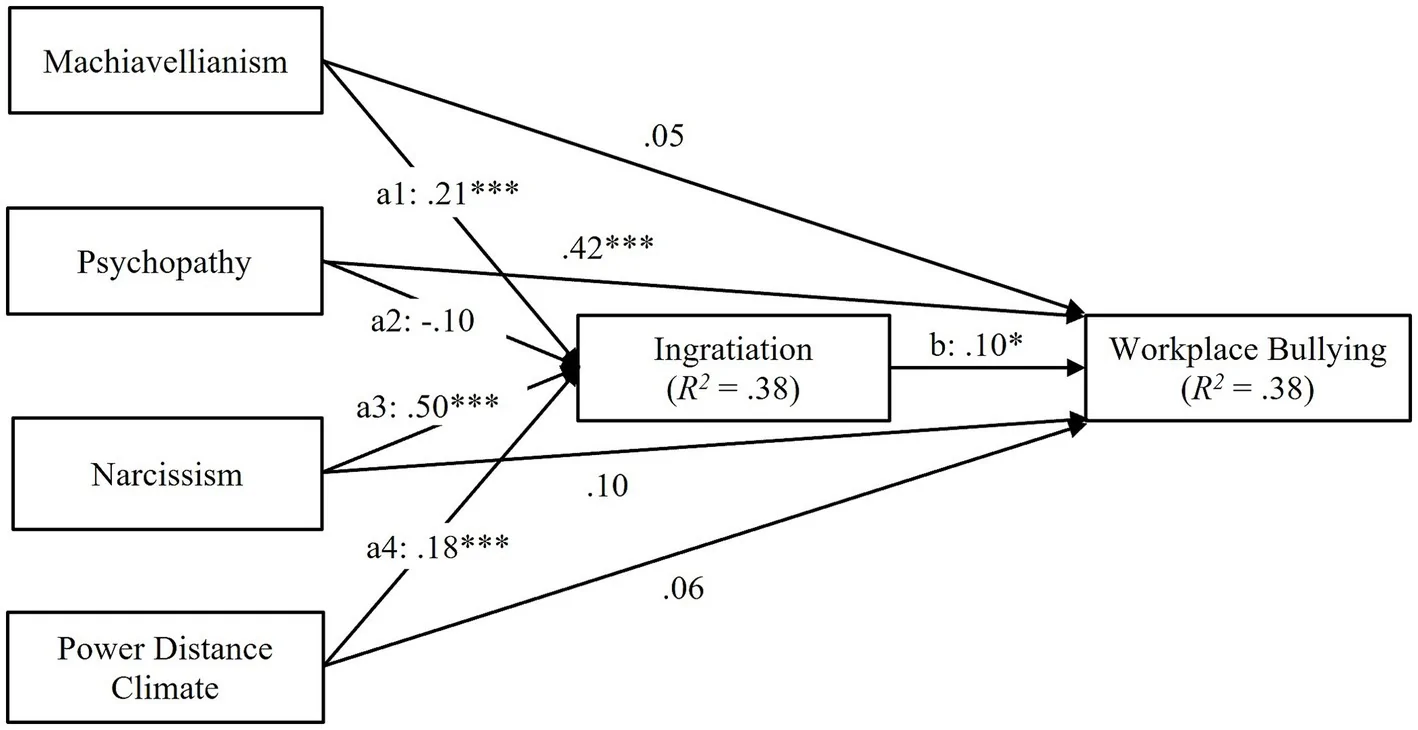
Structural equation model of the proposed relationships among the study variables. Standardized path coefficients are presented. Paths used to estimate indirect effects are labeled (e.g., a1, b1) and correspond to the indirect effects reported in Table 6. Control variables were included in the analyses but are omitted from the figure for clarity.

**Table 6:** Indirect effects of Dark Triad traits on workplace bullying via ingratiation.

Indirect effect	*β*	*B*	SE	95%CI:	
				Lower	Upper
a1*b	0.02	0.05	0.03	0.001	0.11
a2*b	−0.01	−0.02	0.02	−0.08	0.01
a3*b	0.05	0.12	0.06	0.003	0.25
a4*b	0.02	0.05	0.03	0.001	0.12

## 4 Discussion

This study examined the effect of the Dark Triad and power distance climate on workplace deviance. Specifically, from the viewpoint of Trait Activation Theory we examined whether the relationship between the Dark Triad and workplace deviance changed depending on power distance climate. In addition to examining interactions, this study investigated the role of ingratiation as another mediating process in the effect of the Dark Triad and power distance climate on deviant workplace behavior.

### 4.1 Interaction between dark triad and power distance climate on workplace deviance

Based on the results of the interaction between the Dark Triad and power distance climate on workplace deviance, the main effect of psychopathy and power distance climate on both workplace bullying and CWB was significant. The main effect of Machiavellianism was significant only for CWB, whereas the main effect of narcissism was significant only for bullying. Specifically, narcissism was positively associated with workplace bullying under high levels of power distance climate. By contrast, the association became negative under very low levels of power distance climate, suggesting that the expression of narcissistic tendencies may differ across organizational contexts. In addition to workplace bullying, narcissism was negatively associated with CWB under low levels of power distance climate (Johnson-Neyman threshold: centered power distance climate = −6.35) but not with middle and high levels of power distance climate. Contrary to expectations, no interaction was observed between Machiavellianism and power distance climate in workplace deviance. Therefore, these results partially supported Hypothesis 1. In addition to the absence of a significant interaction effect, the main effect of Machiavellianism on deviant behavior was limited. After controlling for other variables, the main effect of Machiavellianism was significant on CWB but not on workplace bullying. The Machiavellian deviance reported in previous research has primarily been observed in conditions where defection leads directly to monetary rewards (e.g., Malesza, 2018; Bereczkei and Czibor, 2014) or where aggression is used to obtain social status (Kerig and Stellwagen, 2010). Therefore, the limited main effect of Machiavellianism observed in this study is consistent with the findings of previous studies. On the other hand, previous studies have reported the interaction of Machiavellianism and power distance climate on workplace deviance, which have primarily focused on organizational contexts characterized by low levels of ethics, justice, and normative regulation (De Hoogh et al., 2021; Fernández-del-Río et al., 2022). In such environments, the likelihood of being sanctioned for deviant behavior may be reduced, thereby increasing the potential benefits associated with workplace deviance for Machiavellian individuals. By contrast, the organizational climate related to power distance examined in the present study, although top-down in nature, reflects an environment characterized by behavioral control and hierarchical relationships (Fukui et al., 2004). Therefore, this may not necessarily represent a context in which the costs associated with workplace deviance are reduced. Consequently, this type of climate may not serve as an organizational cue that facilitates the expression of Machiavellian tendencies through workplace deviance.

In contrast, narcissistic individuals are motivated by the pursuit of superiority, admiration, and positive recognition from others (e.g., Back et al., 2013; Raskin and Hall, 1979). Accordingly, organizational environments that emphasize hierarchy and status differentiation may reinforce narcissists’ grandiose self-views, feelings of superiority, and entitlement. This interpretation is consistent with the present finding that power distance climate strengthens the positive association between narcissism and workplace bullying. Therefore, such environments may increase the motivational relevance of aggressive behaviors toward narcissistic individuals. Interestingly, narcissism was negatively associated with workplace bullying and CWB at low levels of power distance climate. Khatri (2009) noted that in high power distance organizations, decisions are often based on favor and loyalty rather than merit. Thus, the influence of such non-merit considerations may be reduced in perceived environments with lower power distances. Additionally, previous research suggested that individuals with high narcissism may behave in a prosocial manner depending on the situations such as being observed or attracting the attention of others (e.g., Konrath et al., 2016; Song et al., 2024). Taken together, these findings suggest that the potential self-enhancement benefits associated with deviant behavior may be diminished in low power distance environments. In turn, narcissistic individuals may be more likely to pursue a favorable self-image through alternative socially acceptable means.

For psychopathy, only the main effect on workplace deviance was significant, as expected. Therefore, the behavioral expression of psychopathy may require fewer contextual triggers than Machiavellianism or narcissism. Contrary to the relatively weak relationships reported by O’Boyle et al. (2012), psychopathy emerged as one of the most consistent predictors of workplace deviance in this study. One possible explanation is that some of the studies included in previous reviews may have focused on the potential adaptive facets of psychopathy (i.e., successful psychopathy; Lilienfeld et al., 2015; Persson and Lilienfeld, 2019) and therefore examined specific occupational groups or organizational positions. In contrast, the present study employed a brief Dark Triad measure (Jones and Paulhus, 2014) in a broad working population and may have captured more maladaptive aspects of psychopathy. These methodological differences may partially account for the stronger and more consistent associations observed between psychopathy and workplace deviance.

Finally, although annual income was measured as a supplementary indicator of adaptive outcomes in this study, no evidence was found to suggest that ingratiation was associated with income, nor was there any interaction effect between the Dark Triad and power distance climate. However, narcissism showed a positive but weak association with annual income. These findings are consistent with prior research reporting associations between narcissism and adaptive indicators in organizational settings, including income (e.g., Leder et al., 2021; Volmer et al., 2016). Future research should conduct a more detailed examination by incorporating other adaptive indices, such as job satisfaction or career success.

### 4.2 The mediating role of ingratiation

In addition to the interaction results, this study showed that Machiavellianism and narcissism were positively associated with ingratiation, which, in turn, was positively associated with workplace bullying. This result supports Hypotheses 2 and Hypothesis 3. However, the indirect effects were generally small; therefore, they should be interpreted as limited rather than dominant mechanisms underlying workplace bullying. While previous research has extensively examined interpersonal strategies associated with the Dark Triad (e.g., Jonason et al., 2010; Jonason et al., 2012), few studies have focused specifically on ingratiation. Given that Machiavellianism is characterized by a pragmatic and strategic interpersonal orientation (Christie and Geis, 1970), a link to ingratiation is theoretically plausible; conversely, since narcissism is characterized by a grandiose self-image (e.g., Back et al., 2013; Raskin and Hall, 1979), one might hypothesize that narcissists would avoid interpersonal strategies that involve flattering others. However, this study demonstrates a positive association between narcissism and ingratiation, suggesting that narcissists may engage in ingratiatory behavior when doing so does not threaten their grandiose self-views and is perceived as beneficial to their own interests. However, the ingratiation measure used in this study also includes self-presentation behaviors (Kumar and Beyerlein, 1991). Therefore, the observed association between narcissism and ingratiation may partly reflect narcissists’ tendency to actively manage favorable impressions of themselves, rather than solely their willingness to flatter others. Future research should examine whether different forms of ingratiation are differentially associated with Dark Triad traits, thereby contributing to a more nuanced understanding of the interpersonal strategies underlying these traits. Furthermore, even when controlling for the effects of the Dark Triad traits, power distance climate tended to be associated with workplace bullying, albeit to a modest extent. This result is broadly consistent with classical findings suggesting that ingratiatory behavior is closely linked to status hierarchies and power structures (e.g., Jones et al., 1963; Kumar and Beyerlein, 1991; Pattanayak et al., 2005), extending these arguments from interpersonal relationships to a broader organizational climate.

Although the direct effect of ingratiation on workplace bullying was weaker than that of psychopathy, the findings suggest that ingratiation mediates the effects of Machiavellianism, narcissism, and power distance climate on workplace bullying. However, the direct effect of ingratiation on workplace bullying was only marginally significant in the regression analysis, whereas the corresponding path was significant in the SEM analysis. Therefore, although the indirect effects through ingratiation were significant, the specific prediction of Hypothesis 3 regarding the direct effect of ingratiation on workplace bullying was not strongly supported, and should be interpreted with caution. Nevertheless, the findings suggest that ingratiation may have a dark side. While ingratiation offers the potential benefit of relatively elevating one’s organizational status (e.g., Higgins et al., 2003; Kumar and Beyerlein, 1991), it may also carry a dark side associated with bullying. However, this study does not clarify the specific mechanisms linking ingratiation to deviant behavior, nor does it determine whether bullying targets subordinates or undermines peers or superiors. Previous research has indicated that ingratiation is positively associated with CWB through resource depletion or emotional exhaustion (Yan et al., 2020). This suggests that bullying may be used as a means of emotional regulation in response to emotional exhaustion associated with ingratiation. However, Yan et al. (2020) also reported that ingratiation was associated with emotional exhaustion under conditions of low power distance orientation within individuals. Given that the Dark Triad traits are generally associated with a stronger emphasis on power-related values (Kajonius et al., 2015), the motivations underlying ingratiatory behavior may differ according to individual dispositions and organizational contexts. Therefore, future research should examine the motivations underlying ingratiatory behavior and clarify how different forms of ingratiation contribute to workplace deviance.

### 4.3 Limitations and future directions

First, this study had some methodological limitations in the study design. Given the cross-sectional nature of the present study, causal relationships among Dark Triad traits, organizational climate, ingratiation, and workplace deviance cannot be established. In addition, although this study relied solely on self-reported measures, the results may have been influenced by factors such as social desirability, self-evaluation bias, or common method variance. Future studies could incorporate objective indicators, such as supervisor or colleague ratings and performance records, to complement self-report measures. In addition, because multiple hypothesis tests were conducted, the possibility of Type I error cannot be entirely ruled out. Future research should examine the robustness and replicability of the present findings using independent samples.

Another limitation is theoretical in nature. Although the present study interpreted the organizational climate scale as reflecting power distance within organizations, it was not directly assessed using an established power distance measure. This limitation should be considered when interpreting the findings. The traditional dimension has been validated with lower job satisfaction, higher levels of psychological distress and depression, and reduced autonomy (Fukui et al., 2004). Thus, these characteristics suggest substantial overlap with organizational environments commonly associated with power distance (e.g., Daniels and Greguras, 2014; Khatri, 2009). Nevertheless, the significant main and interaction effects observed in the present study are broadly consistent with previous findings on power distance, and suggest that organizational climate characterized by power distance-related features may provide a useful avenue for understanding workplace deviance. Moreover, as much of the existing power distance literature has focused on individual-level value orientations, relatively little attention has been paid to the organizational level of power distance (Daniels and Greguras, 2014). Although the present study assessed employees’ perceptions of their organizational environment rather than organizational-level power distance directly, the findings may provide preliminary support for the relevance of organizational manifestations of power distance and may offer useful insights for future research on macro-level power distance processes in organizational settings. Future research should examine whether these findings can be replicated by using established measures of organizational power distance climate. Finally, this study focused primarily on workplace deviance. From a Trait Activation Theory perspective, the present findings indicate that perceived organizational setting influences the behavioral expression of narcissism, whereas similar effects are not observed for Machiavellianism or psychopathy. Future research should examine organizational conditions that may reduce the antisocial expression of these two traits, as well as contexts in which Dark Triad traits may be expressed in more adaptive and socially beneficial ways.

## 5 Conclusion

Overall, the findings suggest that workplace deviance is associated with two complementary processes. Power distance climate may moderate the behavioral expression of the Dark Triad traits, whereas ingratiation may represent an interpersonal factor through which these traits are associated with workplace deviance. By identifying both moderating and mediating processes, this study provides a more nuanced understanding of how dark personality traits and organizational contexts relate to workplace deviance.

The interaction between narcissism and power distance climate in relation to workplace deviance extends research applying Trait Activation Theory to Dark Triad traits by highlighting the potential role of hierarchical organizational contexts. The findings suggest that the association between Dark Triad traits and workplace deviance may vary depending on organizational contexts and may differ across specific Dark Triad traits. Practically, the present findings suggest that workplace deviance may not be explained solely by individual characteristics. Organizational features that are not typically viewed as inherently harmful or unethical, such as hierarchical structures and ingratiatory behaviors toward authority figures, may also represent potential contextual factors associated with workplace deviance.

## Data Availability

The raw data supporting the conclusions of this article will be made available by the authors, without undue reservation.
