# Supplementary material for: How do Dark Triad traits predict workplace deviance? The moderating role of perceived organizational power distance and the mediating role of ingratiation
Source: Front Psychol. 2026 Sep 24;17:1937936. doi: 10.3389/fpsyg.2026.1937936 (PMC13645518; doi:10.3389/fpsyg.2026.1937936)
Supplement: Supplementary file 1 [file Table_1.docx]

Supplementary Material

Supplementary Material includes six tables: Supplementary Tables 1-4 report the results of the primary analyses conducted using the stricter exclusion criteria (*N* = 288, men = 149, mean age = 42.22±10.71) to assess the robustness of the findings. Supplementary Tables 5 and 6 report the factor analytic results and evidence of construct validity for the Japanese version of the Measures of Ingratiating Behavior in Organizational Setting.

**Supplementary Table 1**. Hierarchical regression analyses predicting workplace bullying using the stricter exclusion criteria.

|  | Step 1 | | | | |  | Step 2 | | | | |  | Step 3 | | | | |
| --- | --- | --- | --- | --- | --- | --- | --- | --- | --- | --- | --- | --- | --- | --- | --- | --- | --- |
|  | *β* |  | *B* | *SE* | *sr^2^* |  | *β* |  | *B* | *SE* | *sr^2^* |  | *β* |  | *B* | *SE* | *sr^2^* |
| Gender | -0.26 | *** | -7.46 | 1.66 | 0.07 |  | -0.10 |  | -2.71 | 1.50 | 0.01 |  | -0.07 |  | -2.08 | 1.38 | 0.00 |
| Age | -0.13 | * | -0.18 | 0.08 | 0.01 |  | 0.07 |  | 0.10 | 0.08 | 0.00 |  | 0.04 |  | 0.05 | 0.07 | 0.00 |
| Tenure | -0.12 |  | -1.29 | 0.69 | 0.01 |  | -0.08 |  | -0.84 | 0.59 | 0.01 |  | -0.07 |  | -0.79 | 0.54 | 0.00 |
| Manager | 0.04 |  | 1.41 | 2.24 | 0.00 |  | -0.01 |  | -0.17 | 1.95 | 0.00 |  | 0.03 |  | 1.16 | 1.79 | 0.00 |
| No system | -0.03 |  | -1.00 | 2.06 | 0.00 |  | -0.05 |  | -1.74 | 1.77 | 0.00 |  | -0.04 |  | -1.43 | 1.63 | 0.00 |
| Ingratiation | |  |  |  |  |  | 0.15 | ** | 0.11 | 0.05 | 0.01 |  | 0.08 |  | 0.06 | 0.04 | 0.00 |
| Mac |  |  |  |  |  |  | 0.06 |  | 0.15 | 0.14 | 0.00 |  | 0.02 |  | 0.04 | 0.13 | 0.00 |
| Psy |  |  |  |  |  |  | 0.36 | *** | 0.82 | 0.16 | 0.06 |  | 0.33 | *** | 0.74 | 0.15 | 0.05 |
| Nar |  |  |  |  |  |  | 0.10 |  | 0.24 | 0.16 | 0.01 |  | 0.12 |  | 0.28 | 0.15 | 0.01 |
| PD |  |  |  |  |  |  | 0.10 |  | 0.28 | 0.15 | 0.01 |  | 0.14 | ** | 0.39 | 0.14 | 0.01 |
| Mac*PD |  |  |  |  |  |  |  |  |  |  |  |  | 0.13 | * | 0.05 | 0.02 | 0.01 |
| Psy*PD |  |  |  |  |  |  |  |  |  |  |  |  | 0.07 |  | 0.03 | 0.03 | 0.00 |
| Nar*PD |  |  |  |  |  |  |  |  |  |  |  |  | 0.22 | *** | 0.10 | 0.02 | 0.03 |
| *R^2^* | 0.10 | | | | |  | 0.35 | | | | |  | 0.45 | | | | |
| *ΔR^2^* | 0.10 | | | | |  | 0.25 | | | | |  | 0.11 | | | | |

*Note*. Manager and No system are dummy-coded variables (non-manager was the reference category). Mac = Machiavellianism. Psy = Psychopathy. Nar = Narcissism. PD = Power distance. CWB = Counterproductive work behavior.

**p* < .05, ***p* < .01, ****p* < .001.

**Supplementary Table 2**. Hierarchical regression analyses predicting counterproductive behavior using the stricter exclusion criteria.

|  | Step 1 | | | | |  | Step 2 | | | | |  | Step 3 | | | | |
| --- | --- | --- | --- | --- | --- | --- | --- | --- | --- | --- | --- | --- | --- | --- | --- | --- | --- |
|  | *β* |  | *B* | *SE* | *sr^2^* |  | *β* |  | *B* | *SE* | *sr^2^* |  | *β* |  | *B* | *SE* | *sr^2^* |
| Gender | -0.29 | *** | -9.76 | 2.01 | 0.08 |  | -0.11 | * | -3.83 | 1.80 | 0.01 |  | -0.10 |  | -3.35 | 1.74 | 0.01 |
| Age | -0.04 |  | -0.06 | 0.10 | 0.00 |  | 0.13 | * | 0.20 | 0.09 | 0.01 |  | 0.11 | * | 0.18 | 0.09 | 0.01 |
| Tenure | -0.07 |  | -0.86 | 0.83 | 0.00 |  | -0.01 |  | -0.12 | 0.70 | 0.00 |  | -0.01 |  | -0.12 | 0.68 | 0.00 |
| Manager | 0.06 |  | 2.36 | 2.71 | 0.00 |  | 0.06 |  | 2.54 | 2.33 | 0.00 |  | 0.09 |  | 3.69 | 2.26 | 0.01 |
| No system | -0.05 |  | -1.91 | 2.49 | 0.00 |  | -0.05 |  | -1.92 | 2.11 | 0.00 |  | -0.05 |  | -2.02 | 2.06 | 0.00 |
| Ingratiation | |  |  |  |  |  | 0.01 |  | 0.01 | 0.05 | 0.00 |  | -0.04 |  | -0.04 | 0.05 | 0.00 |
| Mac |  |  |  |  |  |  | 0.21 | *** | 0.61 | 0.17 | 0.03 |  | 0.19 | ** | 0.56 | 0.17 | 0.02 |
| Psy |  |  |  |  |  |  | 0.40 | *** | 1.10 | 0.20 | 0.07 |  | 0.38 | *** | 1.04 | 0.19 | 0.06 |
| Nar |  |  |  |  |  |  | -0.02 |  | -0.05 | 0.19 | 0.00 |  | -0.01 |  | -0.03 | 0.18 | 0.00 |
| PD |  |  |  |  |  |  | 0.12 | * | 0.41 | 0.18 | 0.01 |  | 0.14 | ** | 0.47 | 0.18 | 0.02 |
| Mac*PD |  |  |  |  |  |  |  |  |  |  |  |  | 0.16 | * | 0.07 | 0.03 | 0.01 |
| Psy*PD |  |  |  |  |  |  |  |  |  |  |  |  | -0.03 |  | -0.01 | 0.03 | 0.00 |
| Nar*PD |  |  |  |  |  |  |  |  |  |  |  |  | 0.15 | ** | 0.08 | 0.03 | 0.02 |
| *R^2^* | 0.08 | | | | |  | 0.36 | | | | |  | 0.40 | | | | |
| *ΔR^2^* | 0.08 | | | | |  | 0.27 | | | | |  | 0.05 | | | | |

*Note*. Manager and No system are dummy-coded variables (non-manager was the reference category). Mac = Machiavellianism. Psy = Psychopathy. Nar = Narcissism. PD = Power distance. CWB = Counterproductive work behavior.

**p* < .05, ***p* < .01, ****p* < .001.

**Supplementary Table 3**. Regression analyses predicting ingratiation using the stricter exclusion criteria.

|  | *β* |  | *B* | *SE* | *sr^2^* |
| --- | --- | --- | --- | --- | --- |
| Gender | 0.04 |  | 1.68 | 2.01 | 0.00 |
| Age | -0.15 | ** | -0.26 | 0.10 | 0.02 |
| Tenure | 0.00 |  | 0.03 | 0.79 | 0.00 |
| Manager | 0.07 |  | 3.23 | 2.60 | 0.00 |
| No system | -0.01 |  | -0.21 | 2.37 | 0.00 |
| Mac | 0.26 | *** | 0.84 | 0.18 | 0.05 |
| Psy | -0.09 |  | -0.29 | 0.22 | 0.00 |
| Nar | 0.49 | *** | 1.57 | 0.19 | 0.15 |
| PD | 0.10 | * | 0.40 | 0.20 | 0.01 |
| *R^2^* | 0.36 | | | | |

*Note*. Manager and No system are dummy-coded variables (non-manager was the reference category). Mac = Machiavellianism. Psy = Psychopathy. Nar = Narcissism. PD = Power distance.

**p* < .05, ***p* < .01, ****p* < .001.

**Supplementary Table 4**. SEM indirect effects using the stricter exclusion criteria.

| Indirect Effect | *β* | *B* | *SE* | 95%*CI*: | |
| --- | --- | --- | --- | --- | --- |
|  |  |  |  | *Lower* | *Upper* |
| a1*b | 0.04 | 0.09 | 0.05 | 0.02 | 0.19 |
| a2*b | -0.01 | -0.03 | 0.03 | -0.11 | 0.02 |
| a3*b | 0.07 | 0.17 | 0.07 | 0.04 | 0.33 |
| a4*b | 0.02 | 0.04 | 0.03 | -0.004 | 0.12 |

**Supplementary Table 5**. Factor loadings and inter-factor correlation for the Measures of Ingratiating Behavior in Organizational Setting (ingratiation).

| Factors | | Items | *β* | *B* | *SE* |
| --- | --- | --- | --- | --- | --- |
| 1 | Other enhancement | 1 | .83 | .83 | .02 |
|  |  | 5 | .83 | .83 | .02 |
|  |  | 8 | .74 | .74 | .02 |
|  |  | 9 | .86 | .86 | .01 |
|  |  | 13 | .83 | .83 | .02 |
|  |  | 15 | .86 | .86 | .01 |
|  |  | 17 | .82 | .82 | .02 |
| 2 | Opinion conformity | 2 | .82 | .82 | .02 |
|  |  | 6 | .78 | .78 | .02 |
|  |  | 7 | .85 | .85 | .02 |
|  |  | 10 | .76 | .76 | .02 |
|  |  | 11 | .74 | .74 | .03 |
|  |  | 16 | .82 | .82 | .02 |
|  |  | 18 | .75 | .75 | .02 |
| 3 | Self-presentation | 3 | .86 | .86 | .02 |
|  |  | 4 | .78 | .78 | .02 |
|  |  | 12 | .83 | .83 | .02 |
|  |  | 21 | .85 | .85 | .02 |
| 4 | Favor rendering | 14 | .87 | .87 | .01 |
|  |  | 19 | .82 | .82 | .02 |
|  |  | 20 | .84 | .84 | .02 |
|  |  | 22 | .81 | .81 | .02 |
|  |  | 23 | .82 | .82 | .02 |
|  |  | 24 | .82 | .82 | .02 |
| Inter factor correlation | | |  |  |  |
|  |  | F1 | F2 | F3 | F4 |
|  | F1 | ― |  |  |  |
|  | F2 | .98 | ― |  |  |
|  | F3 | .95 | .94 | ― |  |
|  | F4 | .96 | .95 | .92 | ― |

**Supplementary Table 6**. Cronbach’s alpha and construct validity of the Measures of Ingratiating Behavior in Organizational Setting (ingratiation).

|  | Cronbach's alpha | Networking ability | | Apparent sincerity | | Social astuteness | | Interpersonal influence | | PSI total score | |
| --- | --- | --- | --- | --- | --- | --- | --- | --- | --- | --- | --- |
| Other enhancement | .92 | .68 | *** | .49 | *** | .57 | *** | .53 | *** | .66 | *** |
| Opinion conformity | .89 | .64 | *** | .48 | *** | .56 | *** | .50 | *** | .63 | *** |
| Self-presentation | .87 | .67 | *** | .49 | *** | .57 | *** | .59 | *** | .67 | *** |
| Favor rendering | .90 | .70 | *** | .48 | *** | .56 | *** | .54 | *** | .67 | *** |
| MIBOS total score | .97 | .71 | *** | .51 | *** | .60 | *** | .57 | *** | .70 | *** |

*Note*. MIBOS = Measures of Ingratiating Behavior in Organizational Setting. PSI = Political Skill Inventory.

****p* < .001.

**Supplementary Table 7**. Factor loadings for the Traditional factor of the Organizational Climate Scale (power distance climate).

| Factor | | Items | *β* | *B* | *SE* |
| --- | --- | --- | --- | --- | --- |
| 1 | Traditional  (Power distance climate) | 1 | .60 | .60 | .03 |
|  |  | 2 | .77 | .77 | .02 |
|  |  | 3 | .80 | .80 | .03 |
|  |  | 4 | .52 | .52 | .04 |
|  |  | 5 | .76 | .76 | .03 |
|  |  | 6 | .44 | .44 | .04 |

**Supplementary Table 8**. Factor loadings for the Negative Acts Questionnaire (workplace bullying).

| Factor | | Items | *β* | *B* | *SE* |
| --- | --- | --- | --- | --- | --- |
| 1 | Workplace bullying | 1 | .92 | .92 | .02 |
|  |  | 2 | .95 | .95 | .01 |
|  |  | 3 | .93 | .93 | .01 |
|  |  | 4 | .94 | .94 | .01 |
|  |  | 5 | .91 | .91 | .02 |
|  |  | 6 | .92 | .92 | .02 |
|  |  | 7 | .92 | .92 | .02 |
|  |  | 8 | .92 | .92 | .01 |
|  |  | 9 | .97 | .97 | .01 |
|  |  | 10 | .93 | .93 | .02 |
|  |  | 11 | .93 | .93 | .01 |
|  |  | 12 | .92 | .92 | .02 |
|  |  | 13 | .93 | .93 | .01 |
|  |  | 14 | .94 | .94 | .01 |
|  |  | 15 | .97 | .97 | .01 |
|  |  | 16 | .97 | .97 | .01 |
|  |  | 17 | .95 | .95 | .01 |
|  |  | 18 | .93 | .93 | .02 |
|  |  | 19 | .95 | .95 | .01 |
|  |  | 20 | .91 | .91 | .02 |
|  |  | 21 | .87 | .87 | .02 |
|  |  | 22 | .94 | .94 | .02 |
